# One Health approach for the SDGs achievement

**DOI:** 10.1093/eurpub/ckac129.017

**Published:** 2022-10-25

**Authors:** S Viegas

**Affiliations:** 1 NOVA National School of Public Health, Lisbon, Portugal; 2 Comprehensive Health Research Center, Lisbon, Portugal

## Abstract

One Health is an approach characterized by the integration of human and animal health, plants, and ecosystems and encourages multisectoral and multidisciplinary efforts to achieve optimal levels of health and collaboration among different sectors and scientific areas to address challenging health problems. Through this approach is possible to obtained better results since the actions taken are normally focused to obtained co-benefits in several of the Sustainable Development Goals, including health for all and cost savings. The dissemination of One Health research and experiences is important to raise awareness for this approach. In this workshop, this will be done with the presentation and discussion of several themes where the One Health approach is of most relevance allowing to identify the most relevant barriers and opportunities for integrating One Health more widely.

